# Inhibition of pulmonary β-carotene 15, 15’-oxygenase expression by glucocorticoid involves PPARα

**DOI:** 10.1371/journal.pone.0181466

**Published:** 2017-07-21

**Authors:** Xiaoming Gong, Raju Marisiddaiah, Lewis P. Rubin

**Affiliations:** 1 Department of Pediatrics, Paul L. Foster School of Medicine, Texas Tech University Health Sciences Center, El Paso, Texas, United States of America; 2 All Children’s Research Institute, St. Petersburg, Florida, United States of America; 3 Department of Biomedical Sciences, Paul L. Foster School of Medicine, Texas Tech University Health Sciences Center, El Paso, Texas, United States of America; Universite Clermont Auvergne, FRANCE

## Abstract

β-carotene 15,15’-oxygenase (BCO1) catalyzes the first step in the conversion of dietary provitamin A carotenoids to vitamin A. This enzyme is expressed in a variety of developing and adult tissues, suggesting that its activity may regulate local retinoid synthesis. Vitamin A and related compounds (retinoids) are critical regulators of lung epithelial development, integrity, and injury repair. A balance between the actions of retinoids and glucocorticoids (GCs) promotes normal lung development and, in particular, alveolarization. Alterations in this balance, including vitamin A deficiency and GC excess, contribute to the development of chronic lung disorders. Consequently, we investigated if GCs counteract retinoid effects in alveolar epithelial cells by mechanisms involving BCO1-dependent local vitamin A metabolism. We demonstrate that BCO1 is expressed in human fetal lung tissue and human alveolar epithelial-like A549 cells. Our results indicate A549 cells metabolize β-carotene to retinal and retinoic acid (RA). GCs exposure using dexamethasone (DEX) decreases BCO1 mRNA and protein levels in A549 cells and reduces BCO1 promoter activity via inhibiting peroxisome proliferator-activated receptor γ (PPARγ) DNA binding. DEX also induces expression of PPARα, which in turn most likely causes a decrease in PPARγ/RXRα heterodimer binding to the *bco1* gene promoter and consequent inhibition of *bco1* gene expression. PPARα knockdown with siRNA abolishes DEX-induced suppression of BCO1 expression, confirming the requirement for PPARα in this DEX-mediated BCO1 mechanism. Taken together, these findings provide the first evidence that GCs regulate vitamin A (retinoid) signaling via inhibition of *bco1* gene expression in a PPARα-dependent manner. These results explicate novel aspects of local GC:retinoid interactions that may contribute to alveolar tissue remodeling in chronic lung diseases that affect children and, possibly, adults.

## Introduction

The enzyme β-carotene 15,15’-oxygenase (BCO1) is a member of a large and diverse family of non-heme iron-dependent carotenoid cleavage enzymes. BCO1 catalyzes the symmetrical (central) cleavage of the β-carotene molecule to produce two molecules of retinaldehyde (retinal), which can then be oxidized to a transcriptionally active form, retinoic acid (RA) [1], or be reduced to retinol (vitamin A) [2]. Retinol can undergo retinyl esterification for storage in tissues (*e*.*g*., liver, lung, adipose) [3]. Vitamin A and RA are essential for normal lung growth, differentiation, and maintenance of pulmonary epithelium [4, 5] and have critical functions in alveolar formation and post-injury respiratory lining regeneration [6–8]. RA, in particular, is a regulator of alveologenesis [9, 10]. Similarly, vitamin A deficiency impairs alveolar maintenance in the adult lung [11].

In mammals, pulmonary vitamin A is accumulated in lipid interstitial cells (lipofibroblasts) located in the periacinar fetal lung during late gestation and is mobilized during the perinatal burst in air sac (alveolar) remodeling and differentiation [12]. In humans, fetal lung vitamin A stores increase during the third intrauterine trimester, then level off towards term, and decrease, presumably by retinoid mobilization, in early postnatal life [13]. In parallel fashion, blood vitamin A concentrations are observed to be lower in preterm than in full-term newborns [14] as well as in preterm infants who develop bronchopulmonary dysplasia (BPD) than in those who do not [15]. Neonatal or antenatal vitamin A supplementation either for preterm infants or vitamin A deficient pregnant women improves vitamin A status and pulmonary function and mitigates chronic lung disease [16, 17].

In mammals, preformed vitamin A must be ingested from dietary sources or produced by metabolism of provitamin A carotenoids, principally β-carotene, by the action of the BCO1 [18, 19]. BCO1 is expressed in epithelial cells in various human adult [20, 21] and developing tissues [22]. BCO1-mediated β-carotene cleavage also serves as a back-up pathway for vitamin A synthesis when vitamin A intake is insufficient. In humans, about 55–75% of β-carotene absorbed by the intestine is cleaved in enterocytes; up to 15–30% of absorbed β-carotene remains intact and is delivered to peripheral tissues [23, 24].

Although β-carotene biochemistry has been investigated for several decades, regulation of BCO1 activity in non-enteric tissues is not well understood. In the gastrointestinal tract, BCO1 activity is transcriptionally regulated. In rat and chicken intestine, BCO1 activity and mRNA levels are nutritionally regulated by β-carotene (substrate) and retinoic acid (product) [25] in a typical metabolic negative feedback loop. Intestinal *bco1* gene expression is enhanced by PPARγ [26]. Mouse *bco1* transcription is activated by PPARγ/RXRα heterodimer binding to a *bco1* gene promoter PPAR regulatory element (PPRE) [26]. Gong *et al*. [27] demonstrated that PPARγ transactivation is essential, but not sufficient, for maximally stimulated human BCO1 expression. Unlike the mouse, human augmented *bco1* expression requires cooperation between transcription factor PPARγ and MEF2 binding to their cognate DNA regulatory elements [27].

Glucocorticoids (GCs) are a class of steroid hormones having broad physiological effects. Clinically, GC therapy is used in threatened preterm birth as maternal-to-fetal treatment to reduce incidence and severity of neonatal respiratory distress syndrome. GCs also are an adjunct treatment in preterm infants with chronic lung disease [28] and in children and adults who have respiratory compromise from asthmatic, fibrotic, and inflammatory conditions [29–31].

Nevertheless, although GC and retinoids both promote alveolar type II cell differentiation and lung maturation, chronic GC exposure can inhibit, rather than promote alveologenesis [32]. Moreover, maternal GC treatment is associated with decreased fetal lung vitamin A stores [33]. The mechanisms underlying these GC:retinoid interactions remain uncertain. We hypothesized that: (*a*) BCO1 activity provides a local source for pulmonary retinoid synthesis, and (*b*) GC alters pulmonary effects of vitamin A through regulating BCO1 expression.

In the present study, we have investigated the metabolism of β-carotene by BCO1 and the effect of GC treatment on the BCO1 expression in A549 cells, a human lung adenocarcinoma-derived cell line exhibiting alveolar epithelial properties. We report that human lung tissue and A549 cells express BCO1 and metabolize β-carotene to biologically active RA isomers. GC decreases BCO1 mRNA and protein levels and inhibits BCO1 promoter activity in A549 cells. The molecular mechanism of GC-mediated inhibition of BCO1 involves upregulation of PPARα expression which, in turn, most likely reduces PPARγ/RXRα heterodimer binding to the BCO1 promoter, resulting in inhibition of BCO1 expression. This study provides the first evidence that GCs antagonize vitamin A signaling through suppression of BCO1 expression and defines a PPARα-dependent transcriptional mechanism.

## Materials and methods

### Plasmids and chemicals

The plasmid pGL3-basic (Promega) and constructs for wild type, truncated and mutated pGL3-BCO1-Luciferase (pGL3-BCO1-Luc) reporter genes [Ensembl Gene, ENSG00000135697], pPPRE-tk-Luc reporter gene, the expression vectors for PPARα, β, and γ, RARβ, RXRα, and pCMV-β-Gal have been described previously [27]. The pCMV-BCO1 expression vector was a gift from the late Dr. Stefan Andersson (University of Houston, Houston, Texas). Dexamethasone (DEX, a synthetic glucocorticoid) and forskolin (FSK, a cAMP activator) were purchased from Sigma-Aldrich (St Louis, MO). The PPARα agonist (WY14,643 in DMSO, stock concentration, 5 mM), PPARβ agonist (GW501516 in DMSO, stock concentration, 1 mM) and PPARγ agonist (GW1929 in DMSO, stock concentration, 2 mM) were obtained from Alexis Biochemicals (San Diego, CA).

### Antibodies

The polyclonal antibody to BCO1 was characterized as described previously [34]. Polyclonal antibodies to PPARα (CAT.# sc-9000), PPARβ (CAT.# sc-7197), and PPARγ (CAT.# sc-7196) were obtained from Santa Cruz Biotechnology (Santa Cruz, CA). The monoclonal antibody to β-actin (CAT.# A1978) was from Sigma.

### Cell culture

A549 cells (CAT#CCL-185, ATCC, Manassas, VA), an adenocarcinoma-derived human alveolar epithelial cell line, were cultured in Dulbecco's Modified Eagle's Medium (DMEM) supplemented with penicillin (100 units/ml), streptomycin (100 μg/ml), and 10% fetal bovine serum (HyClone, Logan, UT) at 37°C in a humidified atmosphere of 95% air and 5% CO_2_. The above-mentioned reagents, with the exception of fetal bovine serum, were purchased from Invitrogen (Carlsbad, CA).

### Transfection and luciferase assays

One day before transfection, A549 cells were plated into six-well tissue culture plates at a density of 5 x 10^5^ cells per well. Transfections were performed using Lipofectamin 2000 reagent (Invitrogen) as described previously [27]. Each transfection was performed using 1.0 μg of luciferase reporter gene DNA that contained deletions or site-directed mutations in the *bco1* promoter plus 0.2 μg of a β-galactosidase expression plasmid (pCMV-β-Gal) as an internal control. For co-transfection assays, the total amount of DNA for each transfection was kept constant using a control vector (pcDNA3). Luciferase activity relative to the pGL3-basic control vector was determined after adjustment for β-galactosidase activity. All transfections were performed in triplicate in at least three independent experiments.

### RNA preparation and RT-PCR

One x 10^6^ cells were seeded in 60-mm culture dishes. On the next day, the cells were switched to medium with 10% charcoal-stripped FBS for 24 h, then to fresh medium with 10% FBS and containing different treatments for an additional 24 h. Total RNA was isolated with TRIZOL reagent (Invitrogen) and DNase I treatment or was extracted using the RNA Mini Kit following the manufacturer’s protocol (Qiagen, Cat# 74106). RT-PCR assays were performed with iScript^™^ cDNA Synthesis Kit (Bio-Rad, Hercules, CA). The sense and antisense primers for human BCO1 were 5'-CACAATGGAAAGCAATACCGATATG-3’ and 5’-GCAGCTTTTGGGGATCAGTA-3’. Primers for human PPARα were 5’-ATCGGCGAGGATAGTTCT-3’ and 5’-AATCGCGTTGTGTGACAT-3’ and for human PPARγ were 5’-CAGATCCAGTGGTTGCA-3’ and 5’-GTCAGCGGACTCTGGATT-3’. The level of GAPDH mRNA was used as an internal standard. GAPDH primers were 5’-TGATGACATCAAGAAGGTGGTGAAG-3’ and 5’-TCCTTGGAGGCCATGTAGGCCAT-3’. The amplification process was conducted for 30 cycles of denaturation at 94°C for 1 min, annealing at 52°C for 1 min, and extension at 72°C for 2 min. The RT-PCR products were fractionated on 1.2% agarose gels and photographed using an Alpha-Imager 2000 documentation and analysis system.

### Real time RT-PCR

Quantitative real time PCR was carried out in triplicate using a Model 7500 fast real time PCR system and the TaqMan method (Applied Biosystems, Foster, CA). Primers and probes for human BCO1 (Hs00363176_ml), 18S rRNA (Hs99999901_sl) and GAPDH (Hs02758991_g1) were obtained from Applied Biosystems. Relative fold changes of gene expression compared with the internal controls 18s rRNA and glyceraldehyde-3-phosphate dehydrogenase (GAPDH) were determined using 2^-ΔΔ*Ct*^ method as described by Livak K.J. et al [35]. Briefly, the fold change was calculated as follow: relative fold changes = 2 –^ΔΔCt^, where ΔΔCt = (ΔCt _treated_)– (ΔCt _untreated_), ΔCt = Ct _target_−Ct _18srRNA_ or Ct _GAPDH_.

### RNA blotting

A human 12-lane MTN^®^ blot (multi tissue Northern Blot, CAT. # 7780–1) containing ~ 1 μg of poly(A)^+^ RNA/lane was purchased from Clontech Laboratories Inc. (Palo Alto, CA). A BCO1 antisense complementary RNA (cRNA) probe was synthesized from a unique BCO1 coding region sequence obtained using a PCR amplified template and *in vitro* transcription (Promega, Madison, WI), T7 RNA polymerase and [α-^32^P]UTP (Amersham Biosciences). Unincorporated nucleotides were separated from the RNA probes by affinity chromatography on Elutip columns (Schleicher and Schuell, Keene, NH). Blots were incubated in ExpressHyb^™^ hybridization solution (Clontech Laboratories) for 2 h and hybridized overnight at 68°C with 2 x 10^6^ cpm probe/ml followed by washing. Autoradiography was carried out at -80°C with Hyperfilm MP for 3 days.

### Electrophoretic mobility shift assay (EMSA)

Nuclear extracts were prepared with NE nuclear and cytoplasmic extraction reagent (Sigma) as described previously [27]. Double-stranded oligonucleotides containing sequences from the human BCO1 promoter were synthesized (Operon Biotechnologies, Inc., Huntsville, AL). The sense sequences tested were: wild-type PPRE, 5’-GCTTGGAAATTAACCTTTAACCAAACAT-3’ and mutated PPRE, 5’-GCTTGGAAATTAtgCTTTAtgCAAACAT-3’ (lower case indicates mutated nucleotides). EMSA reaction mixtures (20 μl final volume) included 5–15 μg of nuclear extract, 25 mM HEPES, 100 mM KCl, 0.1% Nonidet P-40 (v/v), 1 mM dithiothreitol, 5% glycerol and 1 μg of poly (dI-dC) (Sigma) as a nonspecific competitor. After incubation for 20 min on ice, 20 fmol of [γ-^32^P]-ATP end-labeled probe was added and the reaction was incubated for 30 min at room temperature. Protein-DNA complexes were separated on 5% non-denaturing polyacrylamide gels in 0.5X TBE (Tris-borate-EDTA buffer) at 25°C. For competition assays, 100-fold molar excess unlabeled double-stranded oligonucleotide competitor was incubated together with the nuclear extract prior to adding the ^32^P-labeled probe.

### RNA interference

*SMART*pool-sequenced small-interference RNA (siRNA) targeting human PPARα (Accession #NM-005036) and si*CONTROL* Non-Targeting Pool (siRNA negative control) (Dharmacon RNA Technologies, Lafayette, CO) were diluted and stored according to the manufacturer’s instructions. Our siRNA *SMAR*Tpool contained four pooled RNA duplexes containing “UU” 3’-overhangs and 5’-phosphate on the antisense strand. A mixture of several siRNAs ensured effective knockdown of target gene expression. Once 50% confluent, A549 cells were transfected with a final concentration of 100 nM *SMART*pool siRNA or the non-targeting pool. After 48 h of transfection, when target proteins were reduced by 70% to 80% as assessed by western blot analysis, cells were treated for another 24 h with DEX, FSK, or vehicle.

### Western blot analysis

Cells were harvested, lysed in 1 ml of ice-cold mammalian protein extraction reagent (M-PER, Pierce, Rockford, IL) containing 1 mM dithiothreitol, 1 mM phenylmethylsulfonyl fluoride and protease inhibitor cocktail (Roche Diagnostics GmbH), and centrifuged at 15,000 x *g* for 10 min. Protein concentrations were determined by BCA protein assay (Pierce); 50 μg aliquots of protein from each treatment were subjected to 10% SDS-PAGE. After electrophoresis, the separated proteins were transferred to Immun-blot^™^ PVDF membranes (Bio-Rad) by semi-dry blotting and were probed with the appropriate antibody followed by incubation with anti-rabbit (CAT.# RPN4301) or anti-mouse IgG (CAT.# RPN4201) conjugated to horseradish peroxidase (Amersham Biosciences, Piscataway, NJ). Polyclonal PPARα, -β, and -γ and RXRα antibodies were used at 1:500 to 1:1000 dilutions in PBS containing 5% non-fat dry milk. Immunoreactive proteins were detected by using ECL^™^ western blotting (Amersham Biosciences, Piscataway, NJ).

### Extraction of carotenoids and retinoids

β-Carotene, its major metabolites, and various retinoids were extracted as previously described with minor modifications [36]. After cell incubations with the indicated concentrations of β-carotene for the indicated times, cell monolayers were placed on ice, medium was removed, and monolayers were washed 3 times with 0.5 ml of 10 mM sodium taurocholate in PBS to remove surface adherent carotenoids. The washed cells were harvested by brief trypsinization, pelleted, homogenized in 0.5 ml ice-cold PBS, and transferred to glass tubes. Aliquots (0.1 ml from 0.2 mM stock) of butylated hydroxytoluene (BHT) and, when required, an internal standard (echinenone) was added to cell homogenates followed by 1.5 ml dichloromethane/methanol (1:2, v/v) and 2 ml hexane. After three rounds of mixing and centrifugation, the resulting upper hexane-dichloromethane layers were combined, dried, re-dissolved in 0.1 mL dichloromethane/methanol (1:4, v/v), and subjected to HPLC. We also measured the β-carotene content of the medium before and after incubations. Sample handling, homogenization, and extraction were carried out in the cold and dim yellow light to minimize β-carotene isomerization and oxidation.

### HPLC analysis of β-carotene and retinoids

β-Carotene and retinoids were analyzed and quantified as described previously with minor modifications [37] using a Shimadzu HPLC system (Model: UFLC, Shimadzu, Kyoto, Japan) equipped with a PDA detector, SPD-M20A monitoring from 210-670nm, a gradient pump system, LC-20AT and LC Solution (Shimadzu) software. β-Carotene and metabolites were separated on a C30 column (5 μm, 4.6 x 150 mm, YMC; Waters) attached to a guard cartridge (5 μm, 4.0 x 20 mm, YMC; Waters). Isocratic separation was performed at a flow rate of 1 ml/min using acetonitrile:methanol:dichloromethane (60:30:10, v/v/v) containing 0.1% ammonium acetate (mobile phase A). β-Carotene was monitored at 450 nm. Retinol, retinoic acid (RA), and retinal were monitored, respectively, at 325, 350 and 376 nm. For specific experiments, the β-carotene cleavage product, retinal, was separated isocratically on a C18 column (5 *μ*m, 4.6 x 150 mm, Biobasic-18, Thermo Scientific) attached to a guard cartridge (5 *μ*m, 4 x 10 mm, Biobasic-18, Thermo Scientific) using acetonitrile:water (90:10, v/v) containing 0.1% ammonium acetate (mobile phase B) at a flow rate of 1 ml/min. Individual peak identities were confirmed by their characteristic absorption spectra and were quantified in reference to the peak area of the respective standard.

### Statistics

Experiments were conducted either in duplicate or in triplicate and all experiments were repeated at least three times. Data are expressed as mean ± SD from at least three experiments. Student's *t* test was used to evaluate statistical significance of differences between two groups. *P* < 0.05 was considered statistically significant.

## Results

### BCO1 expression in human lung tissue and pulmonary alveolar epithelial (A549) cells

**Fig 1:**
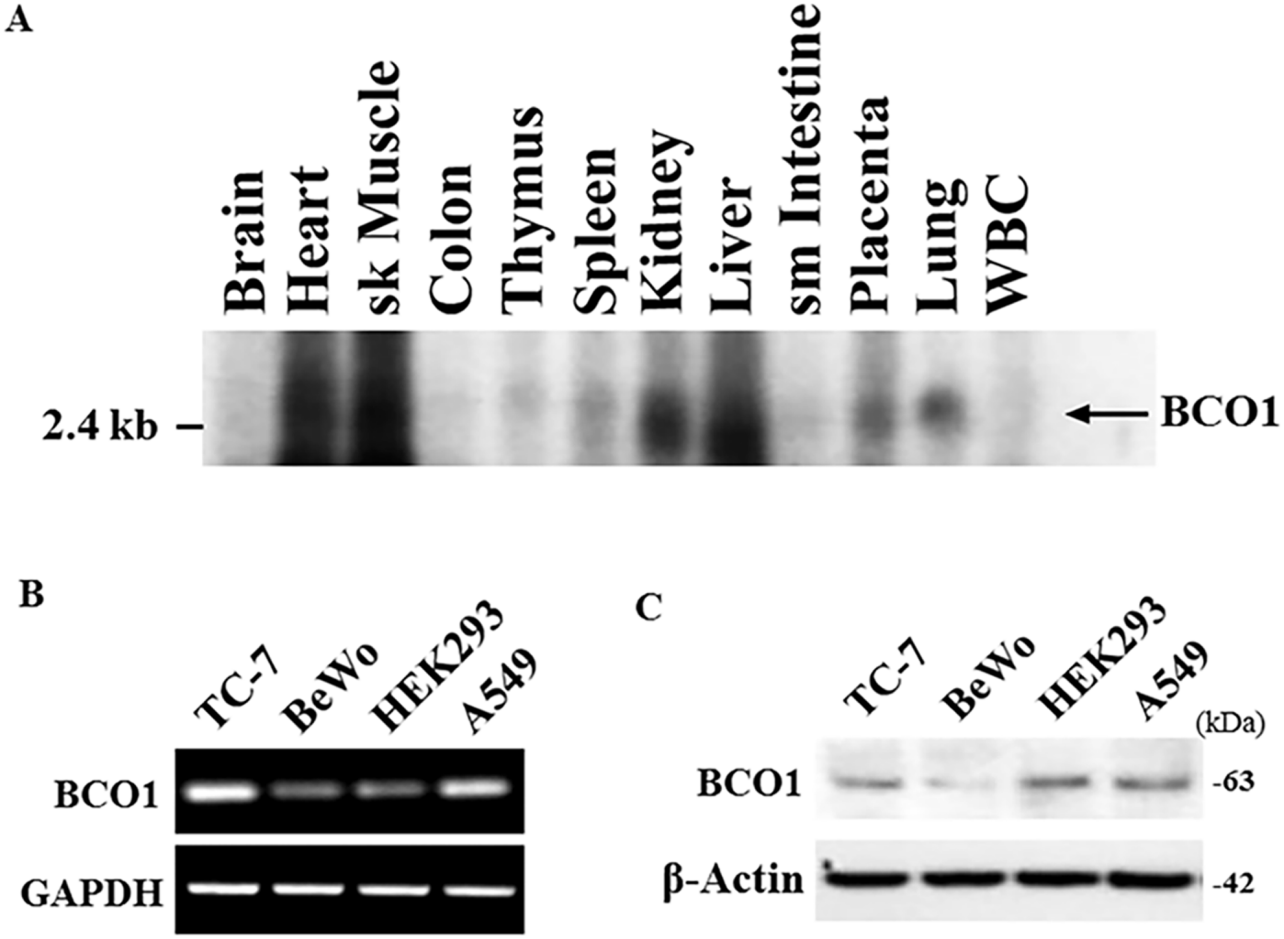
Expression of BCO1 in human lung tissues and a variety of human cell lines. **A**. [α-^32^P]-labeled human BCO1 cRNA probes were hybridized to human poly(A)^+^ RNA (1 μg/lane) from the indicated human tissues. Human tissue filters were exposed to Hyperfilm MP with two intensifying screens at –80°C for 3 days. The position of a molecular size marker is shown at the *left*. **B**. Expression of BCO1 mRNA in human cell lines: intestinal TC7, embryonic kidney HEK 293, placental BeWo, and pulmonary alveolar epithelial A549. Total RNA was isolated and RT-PCR was performed as described in “Materials and Methods”. **C**. Expression of BCO1 protein in the indicated human cell lines: Cell lysates were subjected to western blot analysis using anti-rabbit antibody against the BCO1 protein C-terminal peptide. Equal loading was confirmed by stripping and re-probing the same blot with β-actin antibody.

BCO1 is prominently expressed in human intestine, liver, and kidney [20], consistent with the key enzymatic function converting pro-vitamin A carotenoids to vitamin A. However, in contrast, less information is available on lung BCO1 expression or activity [21, 38]. Consequently, we determined relative abundance of BCO1 mRNA in various human tissues using a sensitive RNA hybridization analysis of human poly(A)+ RNA tissue samples. As shown in Fig 1A, the ~2.5 kb BCO1 mRNA is readily detected in human heart, skeletal muscle, kidney, liver, placenta, and lung.

To determine a range of BCO1 expression, RT-PCR also was carried out in several human cell lines. The TC7 clone of Caco-2 cells exhibits attributes of human small intestinal epithelium including robust BCO1 activity [39]. We also examined placental trophoblastic BeWo cells, the embryonic kidney-derived HEK293 cell line, and pulmonary alveolar epithelial-like A549 cells. As shown in Fig 1B, BCO1 mRNA was detected in these lines, the greatest expression found in TC7 followed by A549. BCO1 message was not detected in several other human mesenchymal and epithelial cell lines (*e*.*g*., ARPE-19, a human retinal pigment epithelial cell line). To verify the presence of BCO1 protein in these cells, we generated an affinity-purified rabbit anti-human BCO1 antibody to a unique sequence in human BCO1 [34] and performed western blot analyses of protein lysates from the cell lines shown in Fig 1B. Consistent with the gene expression (PCR) results, BCO1 protein is present in these lines (Fig 1C).

### Suppression of BCO1 expression and activity by glucocorticoids

**Fig 2:**
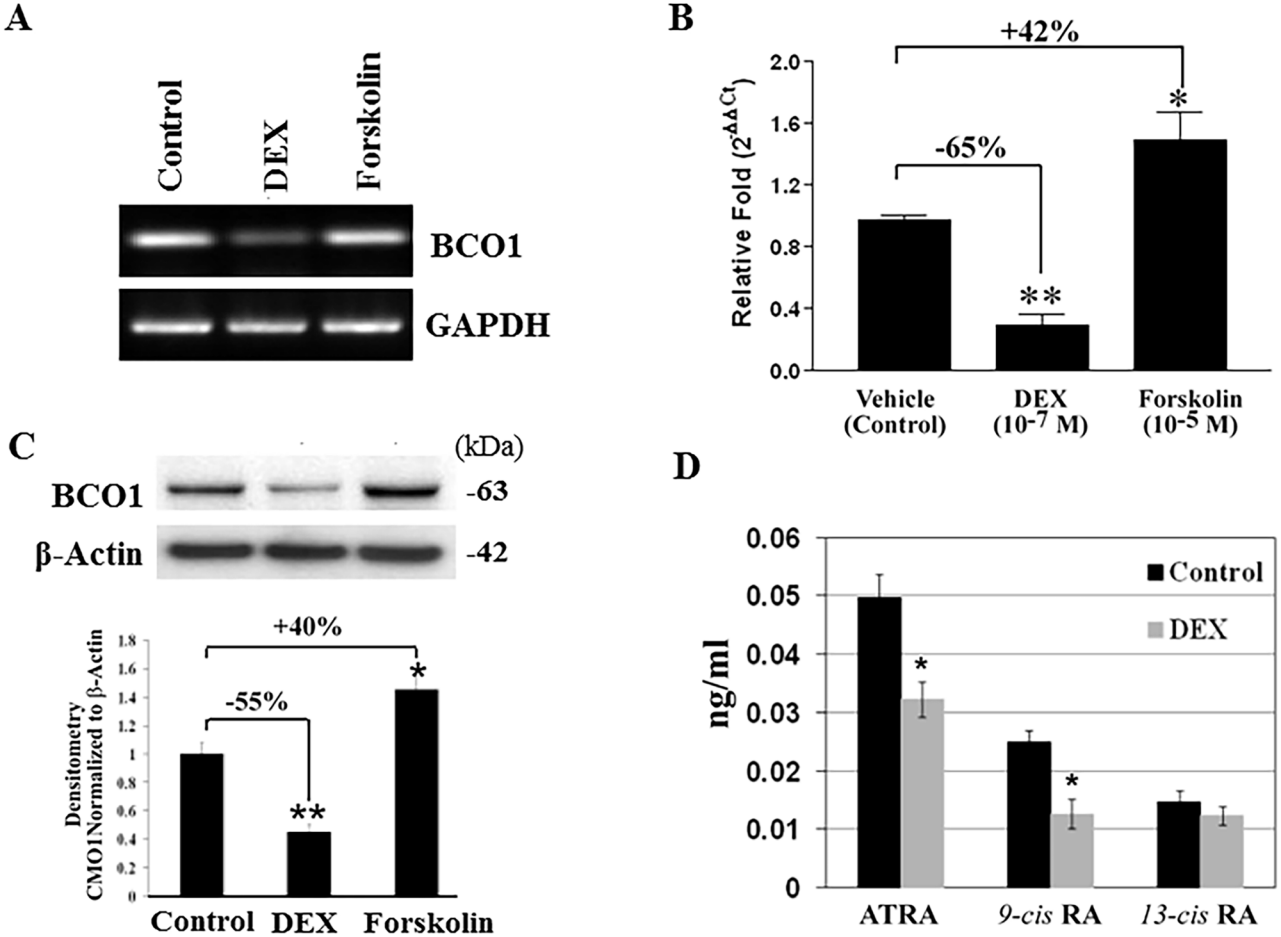
Regulation of BCO1 expression and activity by dexamethasone. **A**. BCO1 mRNA was determined by RT-PCR. Two x 10^6^ cells were plated in 10-cm dishes. After 24 h, cells were treated with DEX (10^−7^ M) or FSK (10^−5^ M) for 24 h. Cells were harvested, total RNA extracted, and RT-PCR performed. GAPDH served as a loading control. **B**. Quantification of BCO1 was examined by real time PCR. Relative fold changes are presented as 2^-ΔΔCt^. Results are presented as means ± S.D. of five independent experiments, each performed in triplicate. * *P*<0.05; ** *P<0*.*01*. **C**. BCO1 protein from each treatment was analyzed by western blot using rabbit anti-BCO1 antibody with β-actin serving as a loading control (*top panel*). The *bottom panel* indicates the adjusted BCO1 levels (mean ± S.D., n = 3). **D**. A549 cells were treated with β-carotene (10^−6^ M) in the absence or presence of DEX (10^−7^ M) for 24 h. Carotenoids and retinoids were extracted from culture media as described in “Materials and Methods”. Retinoic acid isomers were analyzed by HPLC as described above. * *P*<*0.05*; ** *P*<*0.01* compared to respective controls.

Since retinoids (vitamin A) and GCs play interactive roles in lung development and regeneration [40], we asked whether GCs alter carotenoid-to-retinoid metabolism. To this end, human A549 cells were treated with DEX or Forskolin (FSK), an activator of cyclic AMP synthesis, followed by real-time PCR for quantitatively assessing BCO1 mRNA levels and western blot analysis probed with an anti-BCO1 antibody. As shown in Fig 2A and 2B, A549 cell treatment with DEX at a concentration of 10^−7^ M significantly decreased BCO1 mRNA and protein levels (Fig 2C), whereas FSK stimulated BCO1 expression (quantified in Fig 2B and 2C). Consistent with the effects of DEX on BCO1 expression, HPLC analysis showed that production of biologically active retinoic acid (RA) isomers was decreased by DEX (Fig 2D), indicating a BCO1-mediated reduction in β-carotene-to-retinoid activity.

### Reduction of BCO1 promoter activity by dexamethasone

**Fig 3:**
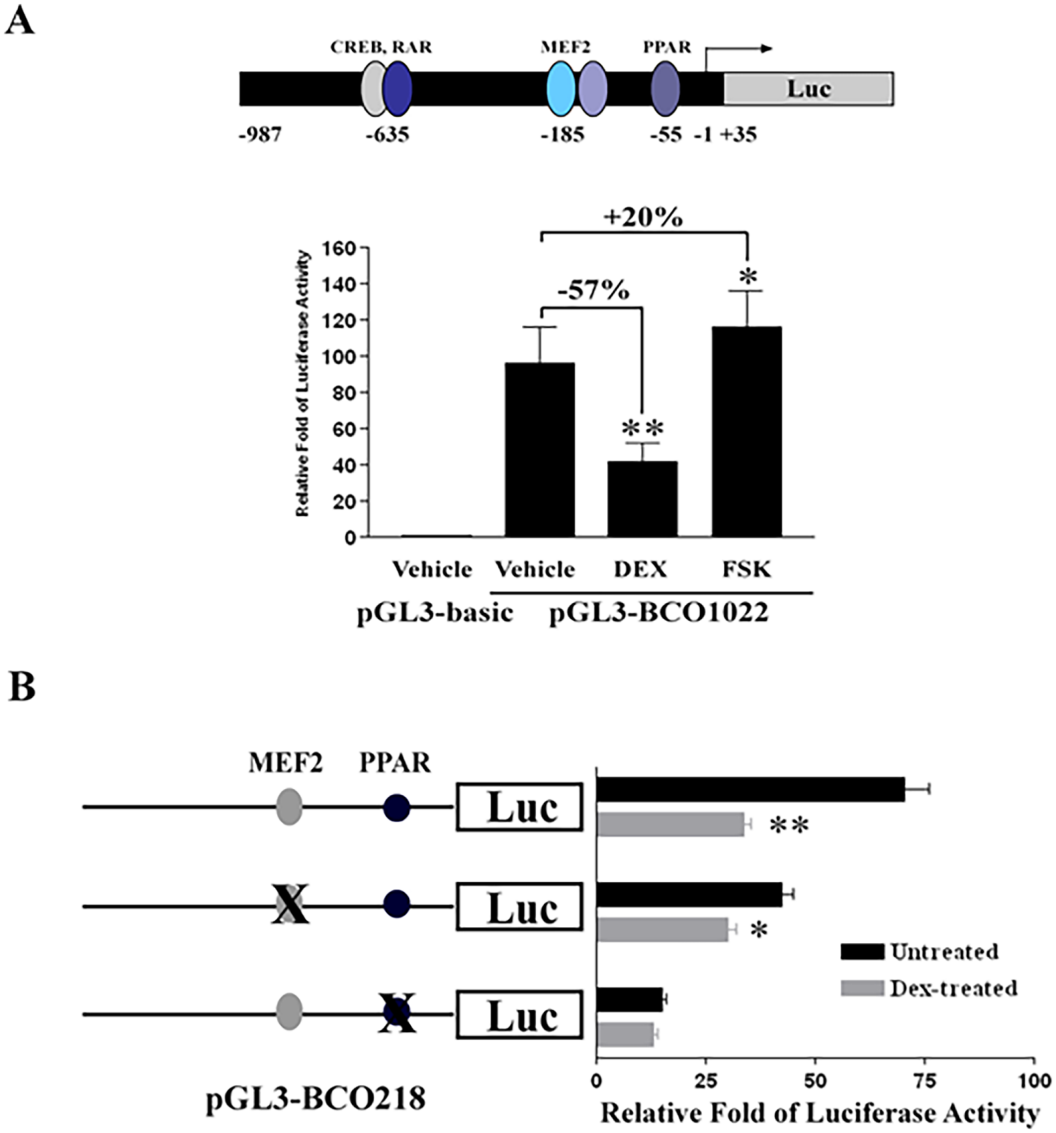
Inhibition of BCO1 promoter activity by dexamethasone. **A**. *Above*, a schematic diagram of the BCO1 promoter reporter gene (pGL3-BCO1022-Luc) showing the location of putative transcription factor binding sites. *Below*, A549 cells were transiently transfected with 1 μg of pGL3-BCO1022-Luc plus 0.2 μg of pCMV-β-Gal vector as a control for transfection efficiency. After 16 h transfection, cells were treated with EtOH (Vehicle), DEX (10^−7^ M) or forskolin (FSK, 10^−5^ M) for 20 h. Luciferase and β-Gal activities were measured and relative-fold luciferase activity (compared to pGL3-Basic transfection) was determined after adjustment for β-Gal activity. Results are presented as means ± S.D. of three independent experiments, each performed in triplicate; * *P*<0.05; ** *P*<0.01. **B**. A549 cells were transiently transfected with 1.0 μg/well of luciferase reporter construct containing either the *bco1* gene proximal 218 bp promoter (pGL3-BCO218-Luc) or with corresponding mutated sequences, as indicated. After transfection for 16 h, cells were treated in the presence or absence of DEX (10^−7^ M) for 20 h. Luciferase and β-Gal activities were measured and relative-fold luciferase activity (compared to pGL3-basic) was determined after adjustment for β-Gal activity. Results shown are means ± S.D. of three or more independent experiments, each performed in triplicate. * *P*< 0.05; ** *P*< 0.01.

Once we established that endogenous BCO1 expression is inhibited by the synthetic glucocorticoid, DEX, we next investigated the possible mechanisms by assessing if the BCO1 promoter reporter construct (pGL3-BCO1022-luc) would similarly behave. A549 cells were transiently transfected with a full-length BCO1 promoter reporter (pGL3-BCO1022) as described previously [27]. Cells were then treated with DEX or FSK for 24 h. As shown in Fig 3A, DEX significantly reduced BCO1 promoter activity to less than half that of vehicle control. The cAMP activator FSK modestly increased BCO1 promoter activity, consistent with the increased direction for BCO1 mRNA, protein, and enzyme activity presented above.

We previously showed the myocyte enhancer factor 2 (MEF2) and PPARγ transcription factors interact to augment human *bco1* gene expression [27]. Although, in contrast, the BCO1 promoter activity is decreased by GCs, no canonical glucocorticoid response elements (GREs) are recognizable in the human *bco1* gene proximal promoter [27], potentially implicating transcriptional mechanisms independent of ligand-activated glucocorticoid receptor (GR):DNA binding. Consequently, upon establishing that DEX can inhibit pGL3-BCO1022 reporter activity, we next tested if this transcriptional regulation is dependent on the MEF2 and/or PPARγ transcription factors by mutating the MEF2 and PPAR binding sites in the BCO1 promoter. A wild-type proximal BCO1 promoter (pGL3-BCO218-luc) construct and two binding site-specific MEF2-mutated or PPRE-mutated reporter constructs were transiently transfected and cells were treated in the presence or absence of DEX (Fig 3B, *left panel*). As shown in Fig 3B (*right panel)*, mutation of the MEF2 binding site decreases BCO1 reporter activity compared with wild-type promoter control in untreated cells. Addition of DEX further inhibits reporter activity, although not sufficiently to account for the bulk of the DEX-mediated inhibition. In contrast, disruption of the BCO1 PPAR binding site abolishes nearly 75% of promoter activation and this effect was independent of the presence of DEX. These results indicate the PPAR binding site in the BCO1 promoter is the principal DNA binding motif mediating DEX-dependent transcriptional repression of BCO1 expression.

### Suppression of PPARγ/RXRα binding to BCO1 promoter by dexamethasone

**Fig 4:**
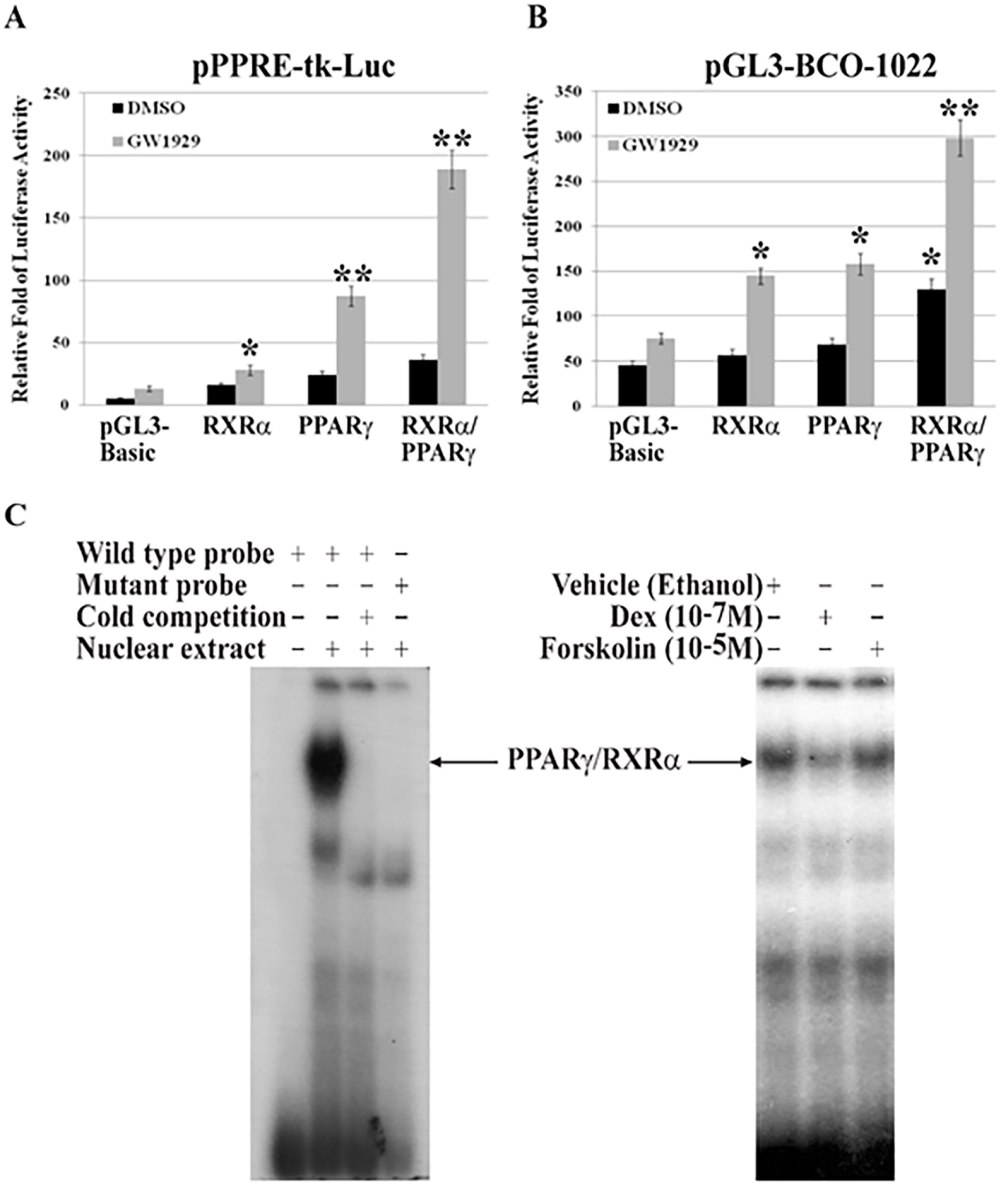
Reduction of the PPARγ/RXRα heterodimer binding to BCO1 promoter by dexamethasone. Luciferase reporters driven by (**A**) pPPRE-tk-Luc or (**B**) pGL3-BCO1022 were transfected into A549 cells using 1.5 μg/well of expression vectors for PPARγ or RXRα, singly or in combination. The total amount of DNA for each transfection was kept constant using control vector DNA (pGL3-basic). After 16 h transfection, cells were incubated with DMSO (control) or the PPARγ agonist GW1929 (2 μM) for 8 h. Luciferase and β-Gal activity were measured and relative-fold luciferase activity was determined after adjusting for β-Gal activity. Results are presented as means ± S.D. of three independent experiments, each performed in triplicate; * *P*<0.05; ** *P*<0.01 vs. pGL3-Basic transfection controls. **C**. EMSA shows nuclear protein-DNA binding to the PPRE binding sequence of the BCO1 promoter. Oligomers were end-labeled with [γ-^32^P]-ATP and incubated with nuclear extracts from A549 cells (see Materials and methods). Addition of 100-fold molar excess of unlabeled competitor oligomers or mutated probes is indicated above each lane (*left panel*). [γ-^32^P]-ATP labeled probe was incubated with nuclear extracts from A549 cells treated for 20 h with Vehicle, DEX, or forskolin as indicated above each lane (*right panel*).

The above findings prompted a closer examination of potential DEX effects on PPARγ/RXRα heterodimer transactivation of the *bco1* gene. In these experiments, we compared activation of a PPAR-specific reporter construct (pPPRE-tk-luc, which is driven by three PPREs, *i*.*e*., 3 x DR1) (Fig 4A) with that of the BCO1 promoter reporter construct (pGL3-BCO1022-Luc) (Fig 4B). A549 cells were transfected with PPARγ and RXRα expression vectors, individually or in combination. PPARγ agonist GW1929 treatment was used to prime the cell signaling pathway. As shown in Fig 4A, the canonical PPRE-luc reporter responded to the PPARγ agonist (GW1929) both in the absence or presence of co-transfected RXRα and PPARγ. Moreover, in the presence of GW1929, co-transfection of PPARγ and RXRα significantly increased PPRE-mediated luciferase activity to a greater extent than did expression of either transcription factor alone. In contrast, the BCO1 promoter (pGL3-BCO1022-luc) required co-transfection of both PPARγ and RXRα to induce maximal reporter activity (Fig 4B). Unlike the situation for the PPRE-tk-luc reporter, BCO1 promoter activity was significantly induced by PPARγ/RXRα, even in the absence of the PPARγ agonist (GW1929), and GW1929 itself significantly augmented BCO1 promoter activity (Fig 4B). These results indicate that PPARγ/RXRα heterodimerization is essential for BCO1 transcriptional activation.

Having demonstrated that PPARγ activity is required for BCO1 expression and DEX-mediated inhibition of BCO1, we next directly examined the effects of DEX and FSK on PPARγ/RXRα heterodimer binding to the BCO1 promoter PPRE site. A549 cells were treated with DEX or FSK for 24 h and nuclear extracts were isolated for electrophoretic mobility shift assay (EMSA). As shown in Fig 4C (*left panel*), the wild type double-stranded probe, when incubated with nuclear extract from A549 cells, forms a single DNA-protein PPARγ/RXRα complex (*2*^*nd*^ *lane*), consistent with our previous findings [27]. The binding specificity and affinity of the PPARγ/RXRα complex were confirmed by competition with excess unlabeled double-stranded wild type oligonucleotide (Fig 4C, *left panel*, *3*^*rd*^ *lane*). Incubation of the nuclear extract with a double-stranded probe containing a mutated PPRE similarly prevented a corresponding formation of the PPARγ/RXRα DNA binding complex. Finally, as shown in Fig 4C (*right panel*), in response to DEX treatment, substantial decrease in binding of the PPARγ/RXRα complex to the PPRE motif is observed. FSK treatment has no significant effect on the binding of the PPARγ/RXRα complex. These results, taken together with the loss of DEX-inducible repression of BCO1 promoter activity when the PPRE site is mutated (Fig 4B), suggest that GC inhibits *bco1* gene expression by reducing the binding of PPARγ/RXRα to the PPRE site in the BCO1 promoter.

### Involvement of PPARα in DEX-mediated inhibition of BCO1 expression

**Fig 5:**
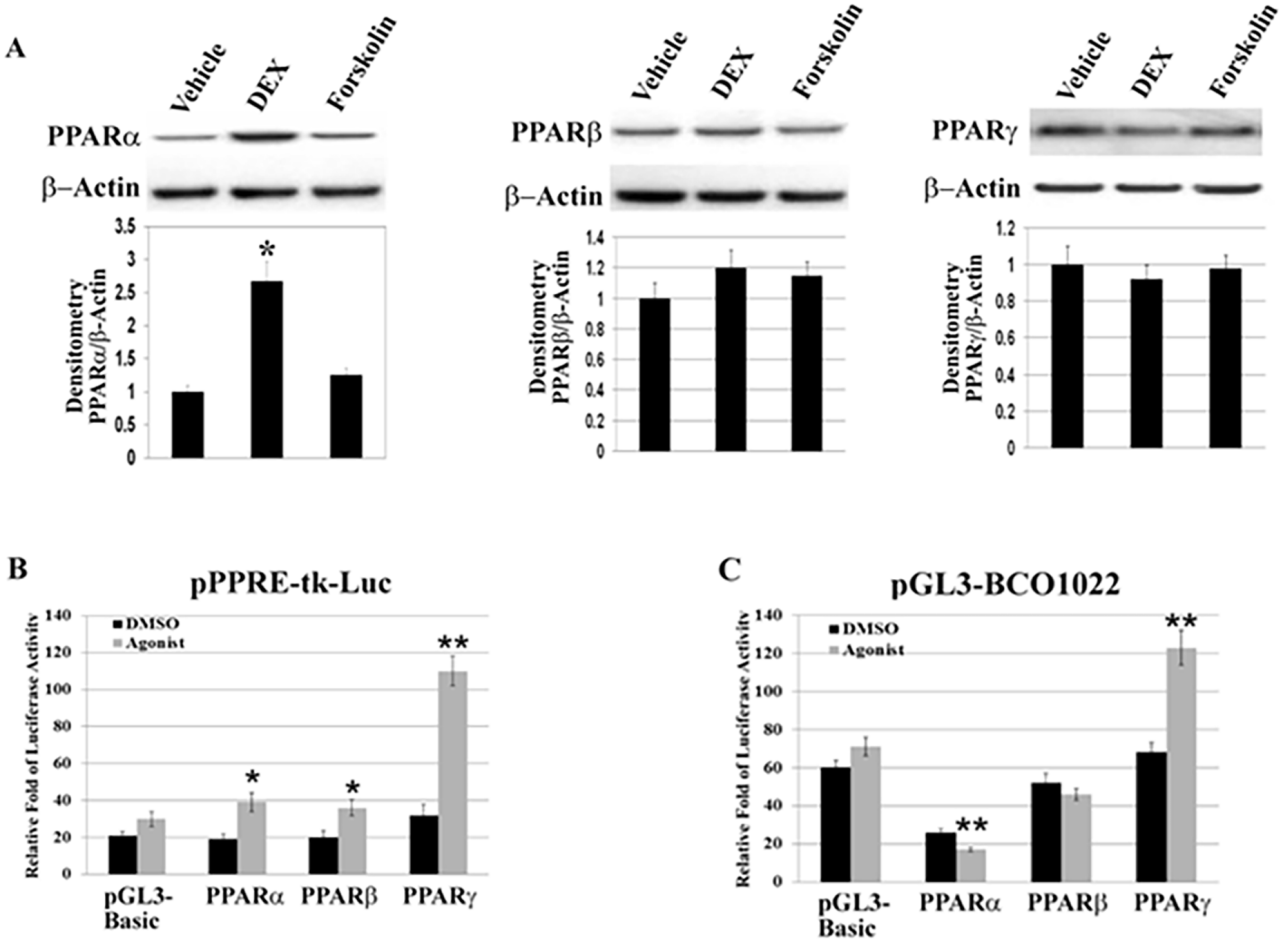
Involvement of PPARα in DEX-mediated inhibition of BCO1 gene expression. **A**. Effect of DEX treatment on expression of PPAR isoforms. A549 cells were treated for 20 h with vehicle (EtOH), DEX (10^−7^ M), or forskolin (10^−5^ M). Cell lysates were subjected to western blot analysis using the indicated antibodies, PPARα (*left panel*), PPARβ (*middle*), and PPARγ (*right*), respectively. Equal loading was confirmed by stripping and reprobing the blots with β-actin antibody. The histogram in Fig. 5A indicates the adjusted PPARα levels (mean ± SD, n = 3). Luciferase reporters driven by pPPRE-tk-Luc reporter (**B**) or pGL3-BCO1022-Luc reporter (**C**) were transiently transfected into A549 cells with expression vectors for PPARα, PPARβ, or PPARγ. The total amount of DNA for each transfection was kept constant using control vector DNA (pGL3-basic). After 16 h transfection, cells were incubated with or without PPAR isoform-specific agonists (5 μM Wy-14,643 for PPARα, 2 μM GW1929 for PPARγ, 1 μM GW501515 for pGL3-basic and PPARβ, respectively) for 8 h. Luciferase and β-Gal activity were measured, and relative-fold of luciferase activity was determined after adjusting for β-Gal activity. Results are presented as means ± S.D. of three independent experiments, each performed in triplicate. * *P*< 0.05; ** *P*< 0.01.

To understand the molecular mechanisms underlying GC-induced inhibition of BCO1 expression, we further examined the effects of DEX on the expression of three PPAR isoforms. As shown in Fig 5A, the PPARα, -β, and -γ isoforms are present in A549 cells. Since the various PPARs may show distinct cellular activities, we determined if DEX treatment alters the PPAR family isoform profile in this system. An unanticipated finding is that DEX treatment of A549 cells leads to a significant increase in PPARα protein levels (Fig 5A, *left panel*). In contrast, there is no significant DEX-related change in the levels of PPARβ or PPARγ (Fig 5A, *middle and right pane*l). Forskolin treatment does not alter the levels of any PPAR isoforms. These data suggest that the DEX-inducible increase in cellular PPARα might be involved in the inhibitory machinery of BCO1 expression.

To verify whether this increase in PPARα protein abundance in response to DEX treatment is essential for the DEX-mediated inhibition of BCO1 expression, we again determined activation profiles for the pPPRE-tk-luc and the full length BCO1 promoter (pGL3-BCO1022-Luc) reporters in A549 cells. These experiments were performed in the presence or absence of PPAR isoform agonists. Cells were transfected with expression vectors for PPARα, -β, and -γ, respectively, and treated with the corresponding PPAR isoform agonists. As proof of principle (Fig 5B), overexpression of each PPAR isoform in the presence of the agonist increases PPRE-mediated luciferase activity, the greatest activity seen for PPARγ (four-fold). We next examined the effect of exogenously expressing each PPAR isoform in A549 cells transfected with the full length BCO1 promoter luciferase reporter gene (pGL3-BCO1022-Luc). As shown in Fig 5C, co-transfection with PPARγ increases BCO1 promoter activity more than two-fold relative to the basal condition. In contrast, PPARα over-expression and agonist activation significantly decrease BCO1 promoter activity. These data indicate that either increased PPARα expression or agonist activation inhibits BCO1 gene transcription.

### Attenuation of PPARα knockdown in DEX-mediated repression of BCO1 expression

**Fig 6:**
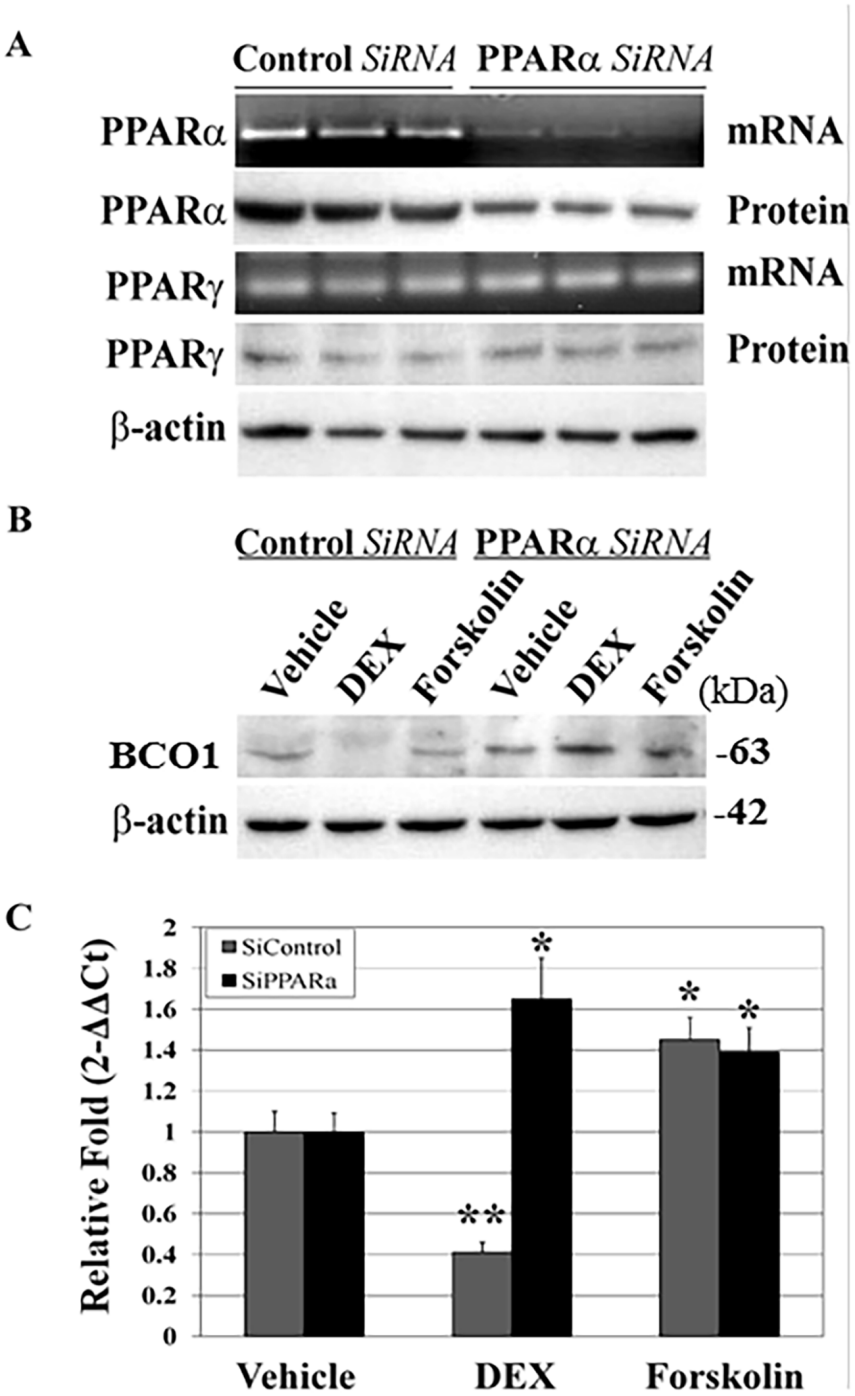
PPARα-dependent inhibition of BCO1 expression by dexamethasone. **A**. Knockdown of endogenous PPARα mRNA and protein by PPARα-specific siRNA: A549 cells were transfected with non-specific control siRNAs or a PPARα-specific siRNA pool. After 72 h transfection, total RNA was isolated and RT-PCR performed as described in Materials and Methods. Cell lysates were subjected to western blot analysis using the indicated antibodies. Equal loading was confirmed by stripping and reprobing with β-actin antibody. **B**. Effects of siRNA-mediated knockdown of PPARα expression on DEX-inhibited BCO1 expression: A549 cells were transfected with non-specific control siRNAs or PPARα-specific siRNAs. After 48 h transfection, cells were treated with vehicle, DEX, or forskolin for 24 h. Equal amounts of cell lysates from each treatment were subjected to western blot analysis using the indicated antibodies. Equal loading was confirmed by stripping and reprobing blots with β-actin antibody. **C**. Effects of siRNA knockdown of PPARα on DEX inhibition of BCO1 mRNA. A549 cells were transfected with the non-specific siRNA or PPARα siRNA. After 48 h transfection, cells were treated with vehicle, DEX, or forskolin for 24 h. BCO1 mRNA expression was quantified by qRT-PCR. Relative fold changes are presented as 2^-ΔΔCt^. Results are presented as means ± S.D. of three independent experiments each performed in triplicate. * *P*< 0.05; ** *P*< 0.01 compared to vehicle control.

In face of the increased PPARα protein levels in response to DEX treatment, we postulated that DEX-induced suppression of BCO1 expression may occur via interaction between PPAR isoforms in a competition mechanism. To validate the importance of PPARα in DEX-induced repression of BCO1 expression, we knocked down PPARα in this cell system. As a result, transfection of *SMART* pool-sequenced siRNA (targeting human PPARα) significantly reduces both PPARα mRNA and protein levels compared to transfection of a si*CONTROL* Non-Targeting pool (siRNA negative control) (Fig 6A). The PPARα siRNA has no effect on PPARγ expression (Fig 6A). Western blot analysis of DEX-mediated BCO1 expression was performed using cells that had been transfected with these siRNA pools for 72 h and were then treated for an additional 24 h with DEX or FSK. We found that DEX-induced inhibition of BCO1 expression is abolished by PPARα siRNA (Fig 6B). There are no significant effects from the non-specific control siRNA pool (siRNA negative control). Consistent with this finding, the control *SiRNA* also does not affect DEX-mediated inhibition of BCO1 mRNA expression. However, the siRNA-specific decrease in PPARα expression results in abrogation of DEX-induced inhibition of BCO1 mRNA expression (Fig 6C). These data further confirm that DEX-mediated inhibition of BCO1 expression is mediated by PPARα.

## Discussion

Local synthesis of vitamin A isomers from β-carotene may be required for certain physiological processes, especially when vitamin A is insufficient. In zebrafish, normal embryogenesis requires BCO1 activity [41]. In mammalian species, BCO1 expression in tissues other than intestine and liver implicates potential enzymatic function in tissue-specific retinoid production, which is important in embryogenesis and lipid metabolism [42–44].

The aim of this study was to determine if glucocorticoid can impact lung retinoid metabolism by regulating provitamin A carotenoid conversion to biologically active retinoids. Our findings show that BCO1 mRNA, protein and enzyme activity are present in human alveolar epithelial A549 cells and that *bco1* gene expression is down-regulated by glucocorticoid.

The presence of BCO1 activity in human alveolar epithelial-derived cells suggests this enzyme can produce retinoids when vitamin A sources may be low, *e*.*g*., during alveolar formation, differentiation, and lung repair. Both retinoids and glucocorticoids are chief regulators of pulmonary growth and function. Since chronic or pharmacological glucocorticoid administration can impair alveolar formation and regeneration [29, 32], it is possible that down-regulation of BCO1 by DEX may antagonize certain retinoid effects, partially by interfering with local retinoid synthesis.

Our experimental strategy sought to elucidate molecular mechanisms underlying a BCO1-mediated interaction between retinoids and glucocorticoids. The results point to specific interactions between PPAR and RXR transcription factors. DEX treatment of A549 cells shifts the PPAR isoform profile toward PPARα from PPARγ and reduces binding of PPARγ/RXRα to the PPRE in the BCO1 promoter.

DEX is known to increase PPARα mRNA and protein levels in liver cells [45, 46], kidney [47], and lung tissue [48, 49]. The current studies indicate DEX treatment enhances PPARα expression which, in turn, inhibits BCO1 expression. In support of PPARα involvement in DEX-mediated repression of BCO1, we showed overexpression of PPARα significantly decreases BCO1 promoter activity (Fig 5C), suggesting ligand-activated PPARα affects the activity of the isoform PPARγ, and thereby inhibiting transcription. At least two possible mechanisms might be involved in the interactions among PPAR family isoforms in DEX-mediated inhibition of BCO1 expression: (*a*) PPARα modulates PPARγ expression in pulmonary epithelial cells, or (*b*) PPARα competes with PPARγ heterodimerization and, consequently, decreases PPARγ/RXRα complex binding to the PPRE in the BCO1 promoter. When we examined the first instance, PPARα knockdown with siRNAs failed to increase PPARγ expression (Fig 6A), which indicates PPARα inhibition of PPARγ activation does not occur through regulation of PPARγ activity. The alternative possibility is increased cellular PPARα levels compete with PPARγ in heterodimerization with RXRα. Our previous EMSA and supershift assay data revealed PPARγ/RXRα heterodimers bind specifically to the PPRE site in the human BCO1 promoter [27]. The current results show DEX treatment of A549 cells most likely reduces PPARγ/RXRα complex binding to the PPRE site in the BCO1 promoter, suggesting increased PPARα may stoichiometrically compete with PPARγ for heterodimer formation with RXRα. It has been noted that the balance of PPAR and RXR plays a role in the heterodimerization of specific PPAR/RXR complex. High PPAR, low RXR or low PPAR, high RXR, lead to less heterodimer formation capable of binding to the PPAR/RXR recognition site in the DNA. On the other hand, competition of PPARs for a limited amount of RXRα to form functional heterodimer may also play a role in both transactivation and transrepression of target genes [50].

In conclusion, we report a molecular mechanism by which DEX-induced PPARα expression negatively affects the activity of PPARγ and downregulates BCO1 gene expression. Loss of BCO1 expression has been linked to obesity and lipid metabolism and lipid accumulation in adipocytes by modulating PPARγ and RAR signaling pathways [51, 52], although remains uncertain if BCO1 affects lipid metabolism in a similar manner in different tissues. DEX-mediated inhibition of BCO1 sheds light on how glucocorticoids can antagonize the effects of retinoids in alveolarization and lipid metabolism during development and in chronic lung diseases. Targeting this pathway could lead to new therapeutic targets for bronchopulmonary dysplasia and other chronic lung diseases that affect both adults and children.
